# Biology, distribution and control of *Anopheles* (*Cellia*) *minimus* in the context of malaria transmission in northeastern India

**DOI:** 10.1186/s13071-016-1878-6

**Published:** 2016-11-15

**Authors:** Vas Dev, Sylvie Manguin

**Affiliations:** 1 National Institute of Malaria Research (Field Station), Guwahati, 781022 Assam India; 2 Institut de Recherche pour le Développement FRANCE (IRD), LIPMC, UMR-MD3, Faculté de Pharmacie, F-34093 Montpellier, France

**Keywords:** *Anopheles minimus*, Malaria, Sibling species, Distribution, Vector bionomics, Insecticide resistance, Vector control, India

## Abstract

Among six dominant mosquito vector species involved in malaria transmission in India, *Anopheles minimus* is a major species in northeast India and held responsible for focal disease outbreaks characterized by high-rise of *Plasmodium falciparum* infections and attributable death cases. It has been now genetically characterized that among the three-member species of the Minimus Complex spread in Asia, *An. minimus* (former species A) is prevalent in India including northeastern states and east-central state of Odisha. It is recorded in all seasons and accounts for perennial transmission evidenced by records of sporozoite infections. This species is highly anthropophilic, and largely endophilic and endophagic, recorded breeding throughout the year in slow flowing seepage water streams. The populations of *An. minimus* in India are reported to be highly diverse indicating population expansion with obvious implications for judicious application of vector control interventions. Given the rapid ecological changes due to deforestation, population migration and expansion and developmental activities, there is scope for further research on the existence of potential additional sibling species within the *An. minimus* complex and bionomics studies on a large geographical scale for species sanitation. For control of vector populations, DDT continues to be applied on account of retaining susceptibility status even after decades of residual spraying. *Anopheles minimus* is a highly adaptive species and requires continuous and sustained efforts for its effective control to check transmission and spread of drug-resistant malaria. *Anopheles minimus* populations are reportedly diminishing in northeastern India whereas it has staged comeback in east-central State of Odisha after decades of disappearance with its eco-biological characteristics intact. It is the high time to siege the opportunity for strengthening interventions against this species for its population diminution to sub-optimal levels for reducing transmission in achieving malaria elimination by target date of 2030.

## Background

In the recent past, with the development of aided tools of molecular systematics, there have been significant advances in our understanding of malaria vector species and disease relationships [1, 2]. With the global efforts for malaria elimination, in-depth study of malaria vectors is regaining its significance for effective vector management. In this drive, India has recently joined the Asia Pacific Malaria Elimination Network (APMEN) with mission to decrease malaria transmission and move into pre-elimination phase by 2017 (www.apmen.org). There are several *Anopheles* species transmitting malaria agents in India and disease epidemiology is complex due to varied ecology and contextual determinants [3, 4]. Among seven main malaria vector taxa in southeast Asia, such as *Anopheles dirus* (*sensu lato*) (*s.l*.), *An. maculatus* (*s.l*.), *An. fluviatilis* (*s.l*.), *An. culicifacies* (*s.l*.), *An. minimus* (*s.l*.), *An. stephensi* and *An. sundaicus* (*s.l*.), *An. minimus* is the major species in the northeastern states of India [5]. During the 1940s, *An. minimus* was widely prevalent and studied for bionomical characteristics and disease transmission relationships in geographical range of its distribution extending from sub-Himalayan foothills of Uttar Pradesh to eastern and northeastern region of India [6–11]. With the advent of DDT and large-scale application for residual spraying during National Malaria Eradication Programme in the 1960s, *An. minimus* was believed to have disappeared from its range [12–15]. Extensive fauna surveys in the Himalayan foothills region did not report *An. minimus* and consequently other prevalent mosquito species were implicated in the continuing disease transmission [16–18]. However, epidemic malaria and emerging drug-resistance, for which northeast India is considered the epicenter, warranted additional investigations to target disease vectors for formulating appropriate containment strategies [19]. In this context, extensive entomological investigations revealed the prevalence of *An. minimus* in northeast India and re-incriminated it by records of sporozoite infections [20–25]. However, there are no records of its return in Terai area of Uttar Pradesh [26], but it has recently resurfaced in eastern state of Odisha (formerly Orissa) after lapse of nearly 45 years of disappearance [27–30]. It has once again been proven unequivocally as the major vector species in the foothill valley areas of eastern and northeast India requiring renewed efforts for its effective control. Given the behavioral characteristics of *An. minimus*, including its plasticity [31], associated to rapid ecological changes owing to human population explosion, development projects, deforestation and human migration affecting mosquito ecology, it was mandated to review its bionomical characteristics and disease relationships. This information is considered important in view of the disappearing malaria and elimination efforts globally. We report the available and most recent information on the systematic position of *An. minimus*, its bionomical characteristics and distribution in India to help formulate species-specific control strategies to reduce transmission in space and time.

### Taxonomy and molecular systematics

*Anopheles minimus* Theobald 1901 (*s.l*.) belongs to the Minimus Subgroup of the Funestus Group, in the Myzomyia Series within the subgenus *Cellia* [32]. It has now been recognized as a species complex comprising three formally named sibling species, including *An. minimus* (*sensu stricto*) (*s.s*.) (former *An. minimus* species A), *An. harrisoni* Harbach & Manguin (former *An. minimus* species C) and *An. yaeyamaensis* Somboon & Harbach (former *An. minimus* species E), with distinct bionomical characteristics and distribution records [33–35]. These three designated species are difficult to distinguish due to overlapping morphological characters, yet these can only be identified reliably by a number of molecular assays [36–38]. Among these, restriction fragment length polymorphism polymerase chain reaction (RFLP-PCR) assay is useful to distinguish in large-scale screening of anopheline fauna, but is more expensive and time consuming [39, 40]. Instead, the allele-specific polymerase chain reaction (AS-PCR) is more convenient, quite reliable and therefore more commonly used for distinguishing *An. minimus* and *An. harrisoni* and closely related sympatric species such as *An. aconitus*, *An. pampanai* and *An. varuna* unequivocally [41, 42].

### Adult morphological distinguishing features

*Anopheles minimus* (*s.l*.) is a small-sized mosquito and can possibly be distinguished from other members of the Funestus Group such as *An. aconitus* and *An. varuna* by a combination of morphological characteristics, such as apical and sub-apical pale bands equal, separated by a dark band; tarsomeres without bands; fringe spot absent on vein-6 wing (anal vein); presence of a presector pale spot and a humeral pale spot on the costa [10, 38]. However, the formal identification of these closely related species cannot rely on morphology only and must be accompanied by the use of an appropriate PCR assay for precise and definite species identification [36].

### Sibling species composition and distribution

**Fig. 1:**
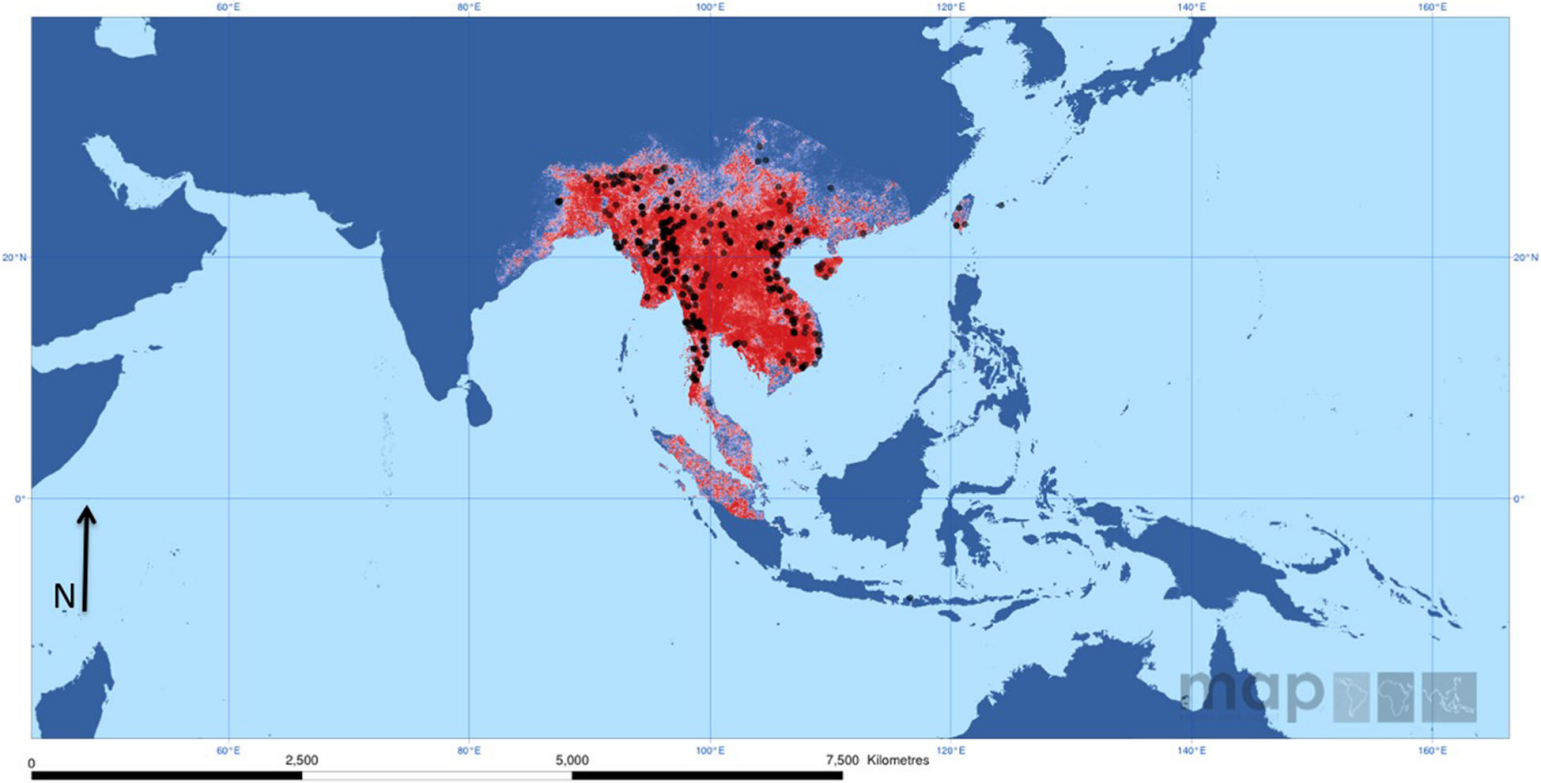
The predicted distribution of *Anopheles* (*Cellia*) *minimus* (*s.l*.) in the world. Red and blue color depicts respectively the high and low probability of occurrence of this complex. Black dots display the sites of data collected. Copyright: Licensed to the Malaria Atlas Project [92] under a Creative Attribution 3.0 License. Citation: Sinka et al. (2011) The dominant *Anopheles* vectors of human malaria in the Asia Pacific region: occurrence data, distribution maps and bionomic précis, *Parasites & Vectors* 2011, 4:89 [2]

**Fig. 2:**
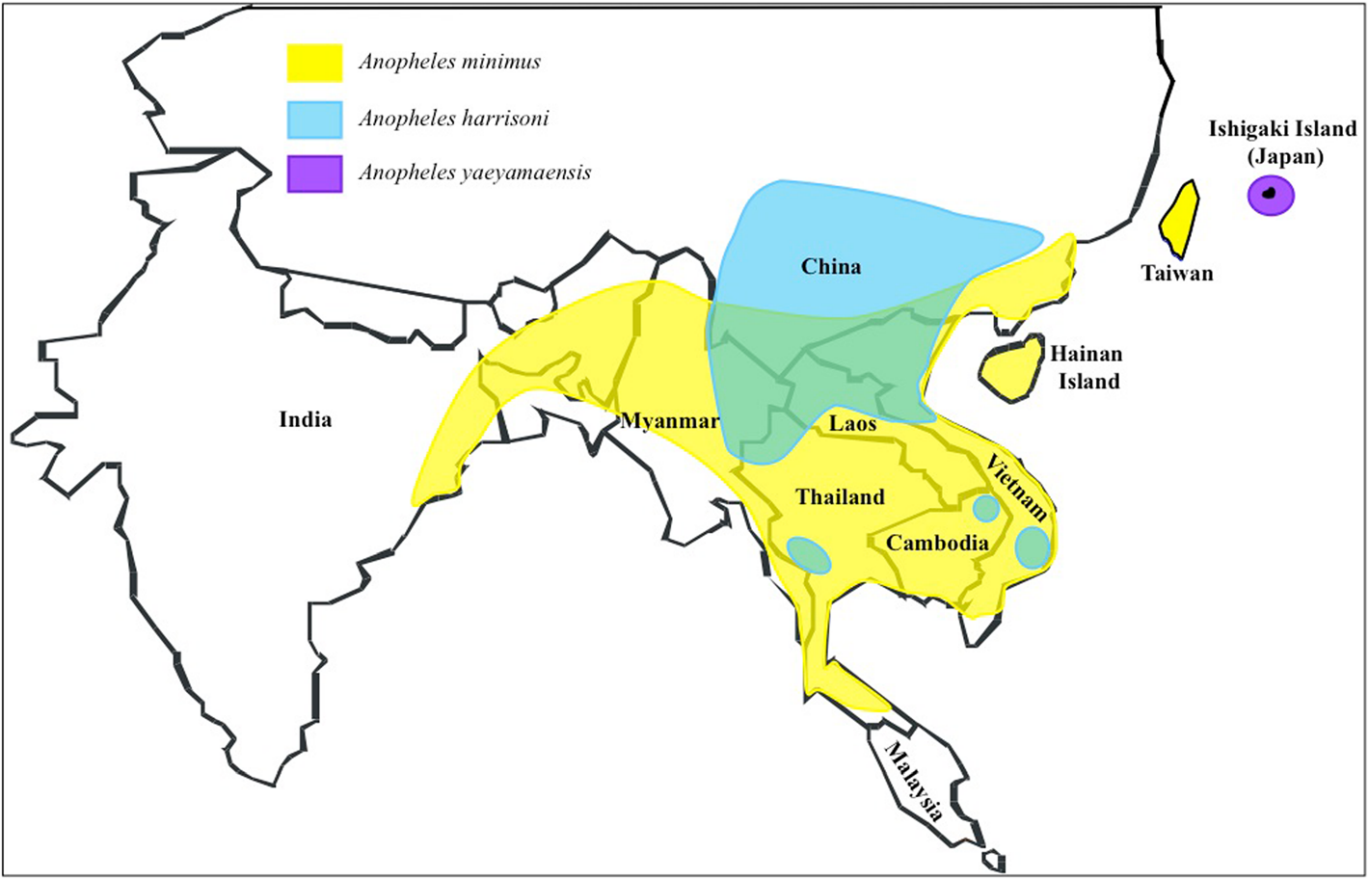
Updated distribution map of sibling species of the *Anopheles minimus* complex in Southeast Asia based on molecular identification. *Anopheles minimus* has wide distribution extending from East India to northeast and eastwards to China including Taiwan, and occurs in sympatry with *An. harrisoni* over a large area in southern China, northern and central Vietnam, northern Laos, and northern and western Thailand. *Anopheles yaeyamaensis* is restricted to Ishigaki Island of the Ryukyu Archipelago in Japan (S. Manguin, original map)

*Anopheles minimus* (*s.l*.) is reported to occur in the Oriental region of countries including India, Myanmar, Thailand, Laos, Cambodia, Vietnam, Southern China comprising Hong Kong, Taiwan and the Ryukyu Islands of Japan [11, 31, 35, 37, 43–45] (Figs. 1 and 2). With molecular identification of the sibling species of the Minimus Complex, the geographical range of each species has now been more detailed [2, 35, 36, 46, 47]. In India, *An. minimus* has a distribution extending from eastern to northeastern regions down to Orissa State and further eastwards to China including Taiwan (Figs. 1 and 2). It occurs in sympatry with *An. harrisoni* over areas in Myanmar, Thailand, Laos, Cambodia, Vietnam and southern China (up to 32.5°N latitude for *An. harrisoni* and up to 24.5°N latitude for *An. minimus*) [31, 36, 40, 43, 46, 48, 49] (Fig. 2). Instead, *An. yaeyamaensis* is exclusively restricted to Ishigaki Island of the Ryukyu Archipelago of Japan (Fig. 2).

In northeast India, *An. minimus* is reported to occur in Assam, Arunachal Pradesh, Meghalaya, Nagaland and Tripura [50, 51] and in eastern State of Odisha [28]. All these populations morphologically identified as *An. minimus* (*s.l*.) were confirmed to be *An. minimus* (*s.s.*) by routinely applied molecular assays including sequencing of the internal transcribed spacer 2 (ITS2) and the D3 domain of 28S rDNA (28S-D3). The prevalence of *An. harrisoni* and *An. yaeyamaensis* could not be established in India. Given the molecular diagnostic assays, *An. minimus*, can now be easily distinguished from other closely related, namely *An. varuna* and *An. fluviatilis* (*s.l*.) having similar geographical range and ecology. In fact, formerly identified populations of *An. fluviatilis* (*s.l*.) from Assam are now genetically characterized to be a hyper-melanic form of *An. minimus* that is prevalent during cooler months [52].

Historically, in India, besides present records of distribution in the eastern and northeastern regions, *An. minimus* was also reported to be prevalent with scattered records of its occurrence in the States of Andhra Pradesh, Tamil Nadu, Kerala and Karnataka [11]. Although these records are dating (1984), there still exists a possibility of its occurrence especially in northern Andhra Pradesh (south of Odisha), given the similar ecology and corridors for transmission in its earlier domains of distribution [53] (Figs. 1 and 2).

### Bionomical characteristics

#### Seasonal prevalence and resting habitat

**Fig. 3:**
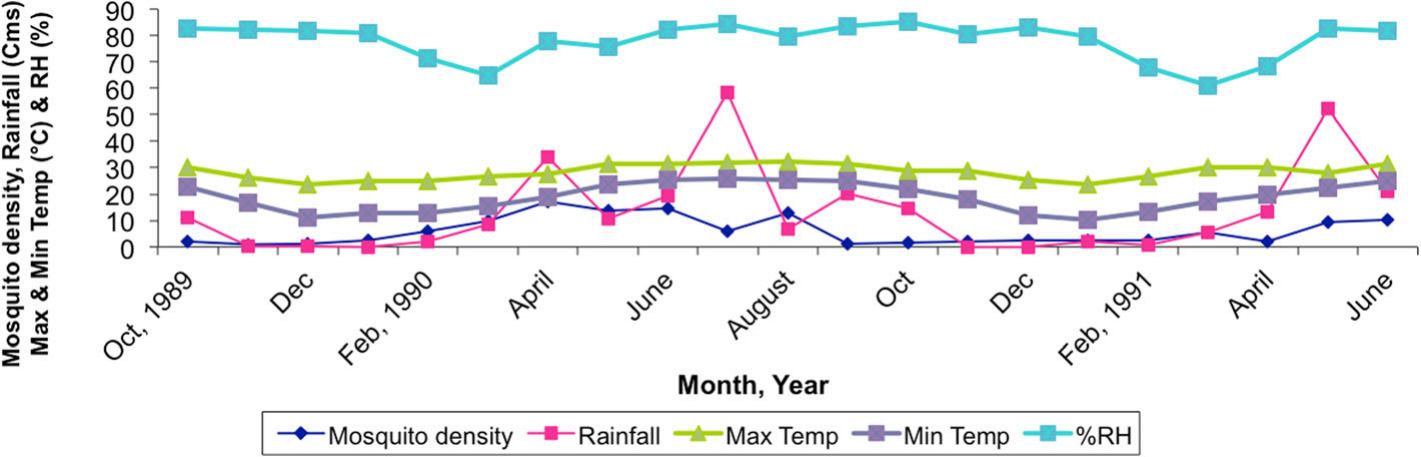
Density of *Anopheles minimus* (number of mosquitoes caught per person hour) and seasonal variations based on meteorological data collected monthly in the Dimoria block of Kamrup district of Assam, northeast India (1989–1991). *Abbreviations*: Cms, centimeters; °C, degree Celsius; RH (%), relative humidity in percent

**Fig. 4:**
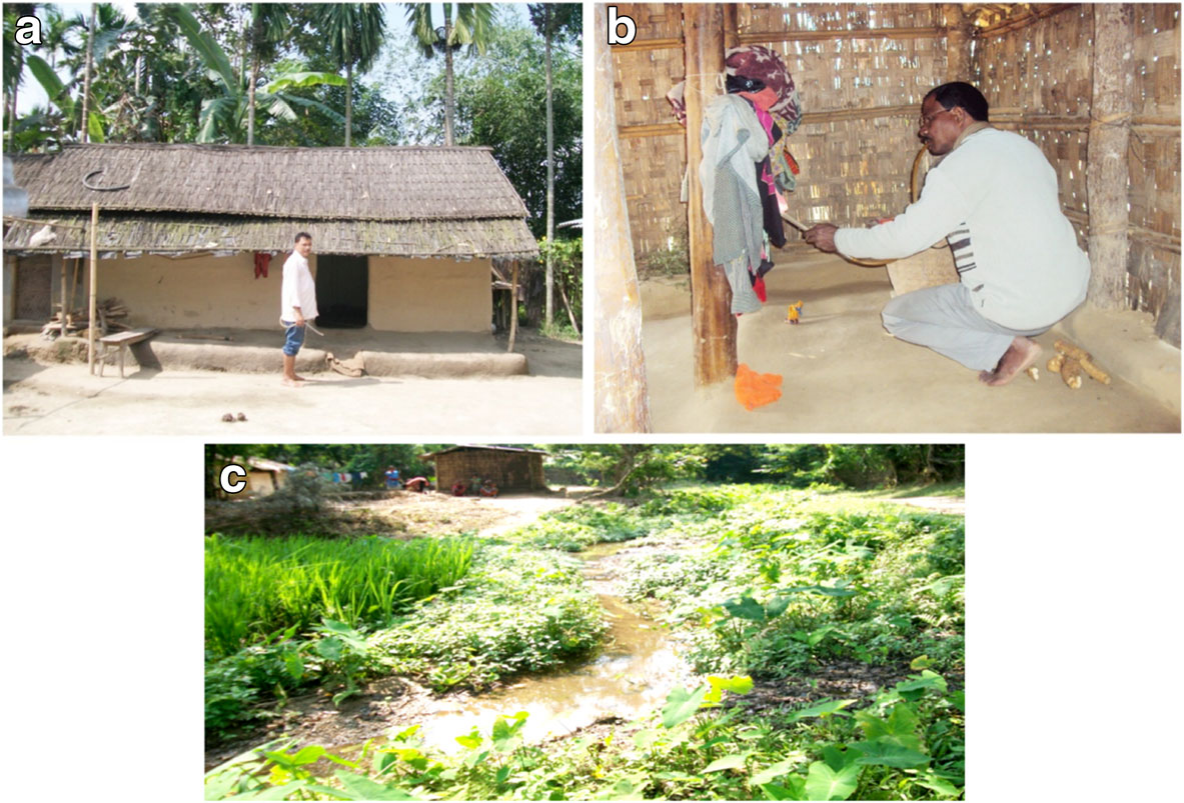
Habitats of *Anopheles minimus*. **a** Typical mud house made of split bamboo with thatched roofing is the preferred housing structure permitting entry of mosquitoes. **b** Hanging articles within house dwellings are ideal resting habitats. **c** Most common breeding habitat of *An. minimus*: seepage water foothill stream with houses located adjacent to breeding resource that are at high risk of malaria

*Anopheles minimus* is characteristically a species of the forested hills and foothill valley areas in most areas of Southeast Asia and India [20, 30, 36, 54]. It is recorded to be prevalent throughout the year at elevations ranging from 100 to 2000 ft above mean sea level (amsl) but its occurrence at higher altitudes up to ~4000 ft (~1000 m) has also been reported [11]. Its relative abundance, however, varied across seasons in different geographical locations [22, 30]. In Assam (Northeast India), its population density appeared rising with increasing temperatures beginning in March (spring season) and peak density was reported in April till August varying from 9.87 to 17.13 specimens per person hour. These were also the months of heavy rainfall (monsoon season) during which maximum and minimum temperatures ranged from 27–32 °C to 19–25 °C, respectively (Fig. 3). For the rest of the year (post-monsoon season), mosquito density remained low and varied from 0.97 to 6.06 per person hour. Instead, in east-central India (Odisha State), peak density was observed during July till October/November coinciding with the wet season and was comparatively low for the rest of the year [30]. In northeast India, *An. minimus* is primarily an endophilic mosquito evidenced by collections of nearly equal proportions of fully fed, semi-gravid/gravid mosquitoes in human dwellings [20]. In contrast, there was indication of exophilic behavior in east-central India marked by lesser proportions of semi-gravid and gravid than fully fed mosquito adults resting indoors [30]. Nevertheless, this species invariably constituted good proportion of indoor resting mosquito collections in non-intervention (unsprayed) human dwellings both in Assam and Odisha [20, 30, 55]. Typically, it is found resting in mud houses/huts made of split bamboo with thatched roofing often adjacent to rice fields/seepage water streams (Fig. 4a and b). Its spatial distribution, however, is highly uneven with houses in closer proximity to breeding habitat (< 1 km) yielding more adults than beyond [56]. In Assam, adult mosquitoes were invariably seen resting on walls in darker corners of the house, hanging clothes, umbrellas and other articles, underneath cots and furniture, etc. (Fig. 4b). In the State of Odisha, however, most adults were observed resting on walls at height of 3–4 ft (1 m) and none on the hanging objects [30]. The species exhibited great degree of behavioral plasticity in response to residual insecticide spray operations and/or introduction of insecticide-treated nets/long-lasting insecticidal nets by changing resting habitat from indoors to outdoors avoiding contact with sprayed/treated surfaces. The mosquito density was reduced to virtually nil in intervention villages [57, 58]. Similar behavioral responses have also been reported in other countries such as Vietnam [48].

### Biting activity and host blood meal preferences

In India, *An. minimus* is primarily an endophagic species having a strong predilection for human host with reported anthropophilic index > 90% across its geographical range both in Odisha and northeastern states [20, 27, 59]. It has a nocturnal biting activity and searched the human host all through the night, beginning at 19:00 h and peaked at midnight onwards till 04:00 h (Table 1). Biting pattern was quite similar across study sites except for northeastern hill state of Mizoram with pronounced biting activity between 20:00 h till midnight (Table 1). Among prevalent *Anopheles* mosquitoes in any given locality, it was the most predominant species in human-landing catches during all months [60]. In the Kamrup District of Assam, mean mosquito-landing rate was 5.82 per person night and varied from 1.00 to 15.83 between months investigated and were the highest during May to July [60]. However, in Odisha, mean mosquito-landing rate indoor and outdoor was 1.76 and 1.71, respectively [29]. In other districts/states of northeast India, the mean mosquito-landing rate per person night between locations varied from 1.66 to 37.50 and seemingly were greatly influenced by interventions, lack of which permitted unusual buildup of vector populations and high human mosquito contact resulting in focal disease outbreaks characterized by high morbidity and attributable death cases [21].

**Table 1:** Records of hourly collections of *Anopheles minimus* of human bait in different districts of northeastern states of India

Study district/State (Study period) [Reference]	No. of mosquitoes collected per person per hour											Total no. of mosquitoes collected (No. of overnight collections)	Mean mosquito landing rate per person/night
		19:00–20:00	20:00–21:00	21:00–22:00	22:00–23:00	23:00–00:00	00:00–01:00	01:00–02:00	02:00–03:00	0300–0400	04:00–05:00		
Kamrup, Assam (June-October, 1988) [20]	Indoor	0.50	1	12	14	16	7	25	25	18	5	123.5 (9)	13.72
	Outdoor	No data											
Sonitpur, Assam (May-June, 1992) [56]	Indoor	2	4	6	1	6	8	11	8	7	0	53 (4)	13.25
	Outdoor	No data											
Darrang, Assam (July-September, 1992) Unpub. obs.^b^	Indoor	7	8	11	13	12	4	10	13	11	3	92 (5)	18.4
	Outdoor	No data											
Morigaon/Assam (July, 1999) [21]	Indoor	0	1	0	3	7	4	6	8	3	3	35 (1)	35
	Outdoor	0	0	0	0	1	4	4	2	6	6	23 (1)	23
Lawngtlai/Mizoram (March, 2005)^a^ Unpub. obs.^b^	Indoor	2	17	10	10	12	7	10	4	3	0	75 (2)	37.50
	Outdoor	No data											
West Garo Hills/Meghalaya (May, 2007) [61]	Indoor	0	0	0	0	0	2	2	0	0	0	4 (1)	4
	Outdoor	0	0	0	0	1	0	1	0	0	0	2 (1)	2
South Tripura/Tripura (June-September, 2012) [50]	Indoor	0	1	5	3	4	6	10	6	2	1	38 (6)	6.33
	Outdoor	0	0	2	0	0	3	3	2	0	0	10 (6)	1.66

^a^Districts reporting focal disease outbreaks

^b^Unpub. obs., unpublished observations

### Vector incrimination, infectivity and inoculation rates

*Anopheles minimus* has been widely incriminated as main malaria vector in India across its geographical range including northeastern states, Bengal and Odisha [10, 11, 27, 30, 60]. In Assam, sporozoite infections were recorded practically all year-round with high seasonal rates of 7 to 8% in post-monsoon month of October [20]. Monthly infection rates varied between seasons but mean infection rates of 2–4% were of common occurrence across study locations [20, 27, 30, 50, 61]. There are records of high mosquito infection rates of > 4%, even up to 15% in disease outbreak areas investigated [10, 11]. Sporozoite infections to the order of 11% were recorded even at high altitude (~2000 ft or 600 m amsl) inclusive of its hyper-melanic population (formerly misidentified as *An. fluviatilis*) during the winter month of December with prevailing minimum and maximum mean temperature of 8.4 °C and 21.4 °C, respectively (unpublished observations). *Anopheles minimus* lived long enough for > 2 weeks evidenced by high parity rate (> 50%) observed in field-caught specimens enabling extrinsic development of infective sporozoites [27, 30, 56]. In Assam, the reported entomologic inoculation rate (EIR = mosquito landing rate × sporozoite infection rate) varied between locations but remained < 1%, representative of low-to-moderate transmission intensities [60]. In Odisha, reported estimated vectorial capacity of *An. minimus* varied from 0.014 to 1.09 for *P. falciparum* and 0.1 to 1.46 for *P. vivax*, respectively, across seasons and was comparable with that of sympatric population of *An. fluviatilis* [27].

### Mosquito flight dispersal and risk factors

It is strongly believed that flight dispersal of *An. minimus* is about one km evidenced by a study of distance from location of breeding habitat, mosquito prevalence and distribution of malaria cases [60]. The risk factor for malaria receptivity in human settlements located nearer to mosquito breeding habitat (≤ 1 km) was estimated to be 10 times greater for having higher parasite rate than those located > 1 km further away. This was further affirmed by large concentration of cases in the focal outbreaks investigated and yield of more cases within the same household suggestive of high vector density and feeding frequency resulting in high morbidity [21, 25].

### Larval breeding ecology

*Anopheles* species are recorded breeding in a variety of aquatic habitats, i.e. paddy fields, ponds, borrow pits, irrigation channels, shallow wells, seepage water streams [36, 62, 63]. Among a variety of aquatic habitats, breeding sites of *An. minimus* in India were reported to occur consistently in seepage water streams all year round in the range of its occurrence [20, 50]. Slow flowing foothill perennial seepage water streams with grassy banks were invariably the specific breeding habitat for *An. minimus* in India, but more globally in Southeast Asia (Fig. 4c) [31, 36, 54]. Occasionally paddy field with perceptible flow of water and shallow wells also contributed to its breeding sites, as well as domestic water tanks commonly found with *An. minimus* larvae in the suburbs of Hanoi [40].

In the pre-DDT era (1940–1942), larval ecology of this species was extensively studied by Muirhead-Thomson in Assam, northeast India for factors governing its abundance [11, 64–67]. The salient findings are summarized below.

#### Selection of breeding places and influence of light and shade

It was established that gravid females of *An. minimus* had some degree of preference for oviposition at specific places rather than random scattering their eggs. Oviposition took place at night and most eggs were laid in first third of the night (69%), 22% in second and 9% in last third of the night. It was concluded that gravid female was strongly attracted by shade, which is normally provided by thick grassy edges of streams while removal of vegetation was observed to be naturalistic control of larvae in a typical breeding habitat. Mosquito perception of light was not acute at low illumination and less likely to be the controlling factor limiting breeding [64].

#### Influence of water movement on selection of breeding places

A series of experiments revealed that contrary to the belief that *An. minimus* breeding was associated with running water, the larvae actually lived in still water shelters provided by grassy vegetation. It was established that gravid females preferred to lay eggs in still water along the edge of drains than in flowing water even at very low velocity of 0.05 ft per second. The larvae were flushed away with water movement exceeding two feet per second [65].

#### Influence of water temperature on choice of breeding places

It was reported that in field conditions the difference in temperatures at night seldom rise 35 °C and did not seem to influence the gravid females for making choice for oviposition. In the laboratory experiments for varied temperatures, there was no marked preference between 23–30 °C; however, gravid females avoided higher temperatures than normally found at night. Egg stage and the first- instar larvae were more resistant to higher temperatures (42 °C) and pupae the least with thermal point < 41 °C. The thermal death point at which no fully grown larvae of *An. minimus* were observed to survive was 41 °C, and was an absolute limiting factor for its breeding in rice fields and borrow pits which typically attain > 41 °C during most of the rainy season. Different developmental stages were observed to grow normally at temperatures ranging between 16–35 °C conditions which are expected to occur in different seasons of the year with evidence for substantial output of females during winter months at lower temperatures [66].

#### Chemical composition of water

Laboratory experiments revealed that gravid *An. minimus* females were very sensitive to pollution by cut vegetation and avoided oviposition. Thus, the high organic content of the water in stagnant paddy fields, water tanks and borrow pits at certain times of the year seemed to explain the mere absence of *An. minimus* breeding in these habitats. However, silt water neither prevented eggs being laid nor the successful growth of the larvae [67].

### Insecticide susceptibility status

In India, *An. minimus* have been repeatedly proven sensitive to DDT over space and time ever since inception of the control programme way back in 1953. Different populations of this species in northeast India were tested to be sensitive to diagnostic concentrations of DDT (4%), as well as malathion (5%), and different pyrethroids, namely alpha-cypermethrin (0.10%), deltamethrin (0.05%) and permethrin (0.75%), employed in the long-lasting insecticidal nets for vector control (Table 2). Similar observations have been reported from the eastern state of Odisha where it has staged comeback after lapse of 45 years [28]. More globally in Southeast Asia, *An. minimus* does not present resistance to pyrethroids, except for northern Vietnam, where resistance to permethrin and lambda-cyhalothrin has been reported [68].

**Table 2:** Insecticide susceptibility status of adult mosquito vector populations of *Anopheles minimus* to diagnostic concentrations of insecticides in northeastern states of India

Study location, district, State [Reference]	Insecticide (Diagnostic concentration)	Study period	No. of mosquitoes exposed (No. of replicates)	No. of mosquitoes knockdown 60 min post-exposure	No. of mosquitoes dead 24 h post-exposure	Mortality (%)	Susceptibility status
Sonapur, Kamrup, Assam (Unpub. obs.)^a^	DDT (4%)	October, 1995	43 (5)	43	43	100	S
Agia, Goalpara, Assam (Unpub. obs.)^a^		October, 1995	13 (1)	13	13	100	S
Sonapur, Kamrup, Assam [92]		July, 1999	80 (4)	80	80	100	S
Sonapur, Kamrup, Assam		October, 2001	80 4)	80	80	100	S
Sonapur, Kamrup, Assam [57]		November, 2005	80 (4)	80	80	100	S
Dalu, West Garo Hills, Meghalaya [61]		June, 2007	30 (2)	30	30	100	S
Bokajan, KarbiAnglong, Assam (Unpub. obs.)^a^		August, 2008	80 (4)	80	80	100	S
Boginadai, Lakhimpur, Assam (Unpub. obs.)^a^		August, 2009	20 (2)	20	20	100	S
Agia, Goalpara, Assam (Unpub. obs.)^a^		October, 2009	44 (4)	44	44	100	S
Amarpur, South Tripura, Tripura (Unpub. obs.)^a^		October, 2010	10 (1)	10	10	100	S
Sidli, Chirang, Assam (Unpub. obs.)^a^		October, 2012	24 (2)	24	24	100	S
Silachari, South Tripura, Tripura [50]		September, 2012	40 (4)	40	40	100	S
Sonapur, Kamrup, Assam [93]	Malathion (5%)	July, 1999	60 (3)	60	60	100	S
Sonapur, Kamrup, Assam [57]	Malathion (5%)	November, 2005	60 (3)	60	60	100	S
Sonapur, Kamrup, Assam [57]	Permethrin (0.75%)	November, 2005	40 (2)	40	40	100	S
Sonapur, Kamrup, Assam [57]	Alpha-cypermethrin (0.10%)	August, 2006	80 (4)	80	80	100	S
Sonapur, Kamrup, Assam [57]	Deltamethrin (0.05%)	November, 2005	40 (2)	40	40	100	S

*Abbreviations*: *S* susceptible (mortality in control replicates was < 5%)

^a^Unpub. obs., unpublished observations

### Vector control and impact of interventions

With the established records of observations for susceptibility to DDT (Table 2), it remains the choice insecticide for control of vector populations of *An. minimus.* In the Indian National Vector-Borne Disease Control Programme, two rounds of indoor residual spraying (IRS), at one gm per square meter, are applied annually coinciding with the high transmission season specific to the region. Despite decades of IRS, since 1953, *An. minimus* remained susceptible to DDT by virtue of its physiological resistance (avoidance of resting on sprayed surfaces by inbuilt extraordinary sensory mechanisms resulting in retaining sensitivity status for long periods despite spray operations over years together) and high behavioral plasticity for adaption to the altered ecology. For monitoring impact of IRS of DDT, the monthly follow up investigations in malaria endemic villages of Assam, during 2001–2002, revealed that *An. minimus* mosquitoes avoided resting on indoor sprayed surfaces until 16 weeks post-spray before its re-appearance in the villages investigated (unpublished observations). Similarly, in the beneficiary population groups receiving long-lasting insecticidal nets (LLIN), *An. minimus* mosquitoes were not seen resting indoor human dwellings even after three years of continuous use of initial distribution. It was observed that LLIN-based intervention not only deterred entry of *An. minimus* species, but also served as personal guard against infective mosquito bites corroborated by data on human mosquito landing catches and declining trends of malaria transmission [57].

### Population diminution and ecological succession

With the introduction of pyrethroid impregnation of community-owned mosquito nets and mass scale distribution of pyrethroid coated/incorporated long-lasting insecticidal nets (LLINs) in communities at high malaria risk, *An. minimus* populations are once again fast diminishing in erstwhile range of this anthropophilic species [69]. The population density is getting scarce presently restricted to isolated far off/inaccessible villages left without intervention for years together. The niche thus vacated is being accessed by *An. culicifacies* (*s.l*.) populations, which are much tolerant to multiple insecticides posing a new challenge for effective control and associated malaria transmission [70]. *Anopheles culicifacies* (*s.l*.) is fast invading degraded forests of the eastern and northeastern states of the country formerly domain of *An. minimus* and *An. baimaii* (Dirus Complex) [71, 72]. In Assam, besides the sibling species ‘B’, a poor to non-malaria vector, sibling species ‘A’ and ‘C’ of *An. culicifacies* are also observed to occur and recorded breeding in seepage water streams sharing breeding habitats with *An. minimus* (Nanda personal communication). In Odisha, recent findings revealed invasion by *An. culicifacies* sibling species ‘A’, ‘D’ and ‘E’ (the latter species being the most efficient vector of the complex), in addition to prevalent species ‘B’ and ‘C’ amounting to added transmission [73, 74]. It was observed that given the population diminution in human dwellings (indoors), *An. minimus* continued to breed in seepage water streams but adult mosquitoes had shifted resting habitat outdoors (Dev, unpublished observations).

### Disease transmission relationships

**Fig. 5:**
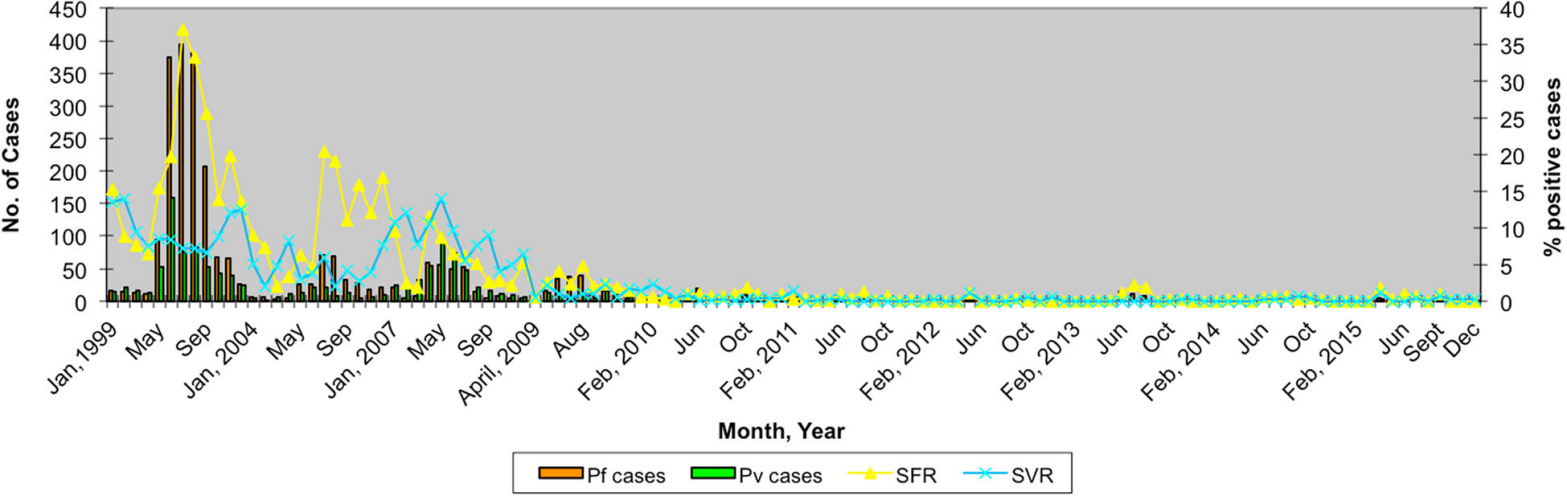
Reducing trends of malaria transmission for monthly data of *Plasmodium falciparum* (Pf) and *P. vivax* (Pv) positive cases and percentages of smear positive cases of *P. falciparum* (SFR) and *P. vivax* (SVR) based on malaria endemic Dimoria block of Kamrup district of Assam, northeast India (1999–2015)

In most parts of northeast India, *An. minimus* was proven unequivocally the major vector responsible for maintaining endemic malaria in foothill valley areas/rice agro-ecosystem studded with criss-crossing perennial seepage water streams providing breeding grounds. In the forest fringe villages, it supplements *An. dirus* (*s.l*.) (*An. baimaii*) and *An. fluviatilis,* transmitting malaria agents in northeast and Odisha respectively, playing predominant role [25, 27]. It is held responsible for focal disease outbreaks characterized by high parasite rates for *P. falciparum* infection (the predominant infection) and associated mortality [21, 56, 75, 76]. In the disease outbreaks investigated, *An. minimus* mosquitoes were invariably observed to be widely abundant and incriminated as vectors. In malaria endemic blocks, the buildup of mosquito vector density preceded that of peak malaria transmission season commencing April/May till September/October corresponding to the wet season [22]. For the rest of the year (dry season), even though vector density remained low, it was responsible for perennial transmission by records of new cases and concurrent sporozoite infections [20]. The high rise in *P. falciparum* cases was observed to be significantly correlated with entomological inoculation rate by *An. minimus* [60]. The buildup of vector populations was largely attributed to inadequate control intervention for the past few years resulting in high infectivity and human-mosquito contact. With the renewed political commitment for strengthening interventions for vector control, prioritizing high-risk blocks of northeastern states and consequent diminishing populations of *An. minimus*, malaria transmission levels were also seen clearly reducing by each passing year formerly intractable (Fig. 5) [77, 78].

### Colonization

Repeated attempts to colonize *An. minimus* in India failed due to poor adult survival and for lack of oviposition in the laboratory conditions. There is an obvious need to colonize this species for better understanding population genetics and its biological characteristics. Attempts to colonize *An. minimus* in the laboratory have, however, been successful based on forced mating technique, but larval development period was much too long lasting 26 days calling for refinements in methodology [79]. Since then, significant improvements in establishing and maintaining colonies of *An. minimus* have been reported in Thailand [80–83]*.*

### Priority areas of research

It is clearly evident that *An. minimus* is a highly adaptive mosquito species with a capacity to survive in varied environments and return to its original habitat after decades of disappearance [28, 31]. It is indeed a dreaded vector species of human malaria in India and Southeast Asia, in areas of its influence. However, there is paucity of data on the distribution and prevalence of *An. minimus* populations (hyper-melanic form in particular) and associated transmission intensities in relation to different elevations from sea level. Given the climate change there exist possibilities of increased transmission windows and disease expansion to higher altitudes for its ability to survive in colder climates [84, 85]. In northeast region of India, even though morphologically identified populations of *An. fluviatilis* has now been molecularly recognized as hyper-melanic population of *An. minimus* [52], more faunistic searches are warranted to rule out the existence of the *An. fluviatilis* complex of species. In addition, *An. minimus* and its hyper-melanic population (formerly identified as *An. fluviatilis*) were reported to occur in sympatry during cooler months (November-April) and were incriminated in malaria transmission [22]. The populations of *An. minimus* in India have been reported to be highly diverse for nucleotide diversity indicating population expansion and possible sub-structuring with obvious implications for judicious application of control interventions [86, 87]. There is scope of research for potential existence of other sibling species within the *An. minimus* complex, particularly in hilly states of northeast India sharing international border with Myanmar for evidence of outdoor resting populations. More information needs to be elicited on the behavioral characteristics of outdoor resting populations for formulating species-specific appropriate control strategies. Northeast India is observing rapid ecological changes due to deforestation, population migration and developmental activities affecting fauna and flora [88]. The landscape changes warrant continued need to monitor the bionomical characteristics and insecticide susceptibility status of *An. minimus* in the context of population diminution and diminishing malaria transmission in erstwhile areas of high receptivity. There is paucity of data for analyses of mitotic karyotypes, polytene chromosome maps and crossbreeding experiments between varied populations of India including hyper-melanic form, which may be of diagnostic significance. It would be just as important to study population dynamics of member species of the *An. minimus* complex in the bordering districts of countries of Myanmar, Bangladesh and Bhutan for developing cross-border initiative in context of the Asia Pacific Malaria Elimination Network (APMEN). *Anopheles minimus* (*s.l*.) has also been implicated in filarial transmission in Asia [89], but there are no data on record even though both east-central and northeastern region of India are endemic for Bancroftian filariasis [78].

## Conclusions

*Anopheles minimus* is an efficient mosquito vector of importance for its high anthropophilic behavior and high *Plasmodium* infectivity rates defining this species as a major malaria vector in Asia. In northeastern states of India, its populations are presently once again diminishing perhaps retracted from the human habitation to outdoors, but the species has not disappeared for sporadic records of its occurrence in isolated pockets left without interventions for extended periods. It is indeed an invincible mosquito species for its innate ability to adapt to ecological changes and history of disappearance and reappearance after decades [20, 28]. Due to this behavioral characteristic, it requires continuous and sustained efforts for its effective control to check transmission and spread of drug-resistant malaria [90]. Given the susceptibility status to residual insecticides in use, there is need to scale-up interventions including LLINs and/or insecticide residual spray operations ensuring full coverage to keep vector populations at bay for achieving much ambitious goal of malaria elimination [91]. With the reducing disease transmission, India has joined the APMEN for achieving pre-elimination in certain feasible districts/States. It is time to seize opportunity for greater allocation of resources for strengthening interventions in northeast region of India (the corridor for spread of drug-resistant malaria) to sustain the gains paving the way forward for freedom from malaria.

## Abbreviations

APMEN: Asia Pacific Malaria Elimination Network
AS: Allele specific
IRN: Indoor residual spraying
ITS2: Internal transcribed spacer 2
LLIN: Long-lasting insecticidal net
PCR: Polymerase chain reaction
RFLP: Restriction fragment length polymorphism
